# Effect of meteorological factors on influenza-like illness from 2012 to 2015 in Huludao, a northeastern city in China

**DOI:** 10.7717/peerj.6919

**Published:** 2019-05-03

**Authors:** Ying-Long Bai, De-Sheng Huang, Jing Liu, De-Qiang Li, Peng Guan

**Affiliations:** 1 Department of Epidemiology, School of Public Health, China Medical University, Shenyang, Liaoning, China; 2 Department of Child and Adolescent Health, School of Public Health, China Medical University, Shenyang, Liaoning, China; 3 Department of Mathematics, School of Fundamental Sciences, China Medical University, Shenyang, Liaoning, China; 4 Division of Infectious Disease Control, Huludao Municipal Center for Disease Control and Prevention, Huludao, Liaoning, China

**Keywords:** Influenza-like illness, Meteorological factors, Temperature, Sentinel surveillance

## Abstract

**Background:**

This study aims to describe the epidemiological patterns of influenza-like illness (ILI) in Huludao, China and seek scientific evidence on the link of ILI activity with weather factors.

**Methods:**

Surveillance data of ILI cases between January 2012 and December 2015 was collected in Huludao Central Hospital, meteorological data was obtained from the China Meteorological Data Service Center. Generalized additive model (GAM) was used to seek the relationship between the number of ILI cases and the meteorological factors. Multiple Smoothing parameter estimation was made on the basis of Poisson distribution, where the number of weekly ILI cases was treated as response, and the smoothness of weather was treated as covariates. Lag time was determined by the smallest Akaike information criterion (AIC). Smoothing coefficients were estimated for the prediction of the number of ILI cases.

**Results:**

A total of 29, 622 ILI cases were observed during the study period, with children ILI cases constituted 86.77%. The association between ILI activity and meteorological factors varied across different lag periods. The lag time for average air temperature, maximum air temperature, minimum air temperature, vapor pressure and relative humidity were 2, 2, 1, 1 and 0 weeks, respectively. Average air temperature, maximum air temperature, minimum air temperature, vapor pressure and relative humidity could explain 16.5%, 9.5%, 18.0%, 15.9% and 7.7% of the deviance, respectively. Among the temperature indexes, the minimum temperature played the most important role. The number of ILI cases peaked when minimum temperature was around −13 °C in winter and 18 °C in summer. The number of cases peaked when the relative humidity was equal to 43% and then began to decrease with the increase of relative humidity. When the humidity exceeded 76%, the number of ILI cases began to rise.

**Conclusions:**

The present study first analyzed the relationship between meteorological factors and ILI cases with special consideration of the length of lag period in Huludao, China. Low air temperature and low relative humidity (cold and dry weather condition) played a considerable role in the epidemic pattern of ILI cases. The trend of ILI activity could be possibly predicted by the variation of meteorological factors.

## Introduction

Influenza is a legally mandated notifiable disease in China (Yang et al., 2009). The outbreaks or epidemics of influenza can lead to absenteeism and productivity losses; they also pose great challenges to the limited health resources during peak illness periods (Guo et al., 2012; Tsai, Zhou & Kim, 2014). Influenza-like illness (ILI), a medical diagnosis of possible influenza or other illness causing a set of common symptoms such as fever, shivering, chills, malaise, dry cough, loss of appetite, body aches and nausea, typically in connection with a sudden onset of illness, is often adopted as the surrogate of influenza in epidemiological sentinel surveys because it is simple and can be made according to the standardized criteria (Minh An, Ngoc & Nilsson, 2014; Wei et al., 2013). The epidemiological characteristics of ILI cases are useful decision support for understanding the transmission of influenza identifying the appropriate time for influenza vaccination and providing rational estimate for the allocation of health care resources (Schanzer & Schwartz, 2013; Zaman et al., 2009).

The timing of seasonal ILI cases distribution varies widely in the regions of different latitude, indicating the involvement of climatic factors (Axelsen et al., 2014; Hu et al., 2012; Oliveira et al., 2016). There is already convincing evidence of the link between the variations of meteorological factors and influenza epidemics on the basis of ILI sentinel surveillance network (Jusot, Adamou & Collard, 2012). It has been proved that the aerosol spread of influenza virus was dependent upon both ambient relative humidity and temperature in the animal model (Lowen et al., 2007) and it has been proposed that some meteorological factors such as the sunshine or solar radiation could affect the influenza epidemiology through the mediation effect of Vitamin D synthesis on individuals’ immune responses to virus infection (Cannell et al., 2006; Juzeniene et al., 2010; Liu et al., 2006). In the context of that weather forecast is more and more precise, the role of meteorological factors, such as rainfall, average relative humidity and temperature, and their influence on ILI can be better understood for the aim of strengthening influenza control (Li et al., 2013; Roussel et al., 2016). As the climate pattern is region-specific and seasonal influenza epidemic varies across latitude, the influenza control policy should be made on the local basis (Emukule et al., 2016; Mahamat et al., 2013; Nimbalkar & Tripathi, 2016). The present study aimed to describe the epidemiological patterns of ILI in Huludao and seek scientific evidence on the link of ILI activity with meteorological conditions in a national ILI sentinel surveillance site of China in the coastal city with a warm temperate climate.

## Materials & Methods

### Study setting, ILI surveillance data collection and ethical considerations

**Figure 1:**
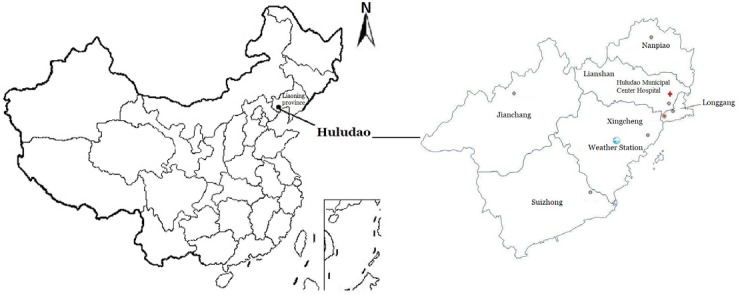
Location of Huludao city which is located in the southwest part of Liaoning province in the northeastern part of China. The boundaries used in this map do not imply the expression of any opinion concerning the legal status of any territory, city or area of its authority or concerning the delimitation of its frontiers and boundaries.

This work was based on the ILI dataset supplied by Huludao Municipal Center for Disease Control and Prevention, which served a total of 2.78 million inhabitants. Huludao is a coastal city (40°N and 120°E) of Liaoning province in the northeastern part of China (Fig. 1) (Guan et al., 2009). The dataset included all ILI cases reported by experienced clinicians in Huludao Municipal Center Hospital on a weekly basis between January 1st, 2012 and December 31st, 2015. A total of 208 weeks’ ILI data was collected, and the start time of the first week is January 2nd, 2012.

Huludao Municipal Center Hospital is one of the China national ILI sentinel surveillance sites (Fig. 1), the number of ILI cases together with their age information and the total visits to outpatient and/or emergency departments of internal medicine and pediatrics were recorded daily and reported weekly through an Internet-based platform maintained by the Chinese Center for Disease Control and Prevention. The surveillance was conducted according to the existing guidelines provided in the 2010 Edition of China National Influenza Surveillance Program (General Office of the Ministry of Health, China, 2010). The standard case definition of ILI was fever (body temperature higher or equal to 38 °C) plus cough or sore throat, in the absence of other alternative diagnoses (Xu et al., 2013; Yang et al., 2009). The data were aggregated by week (Monday to Sunday) and age category.

Research institutional review boards of China Medical University and Huludao Municipal Center for Disease Control and Prevention both determined that the collection of data from ILI cases was part of continuing public health surveillance of a legally mandated notifiable infectious disease and was exempt from institutional review board assessment. All the data were supplied and analyzed in the anonymous format, without any access to the personal identifying information.

### Meteorological data

Daily meteorological data from Xingcheng Weather Station (the closest national meteorological station nested in Huludao city, within 20 kilometres of the population-weighted centre of the city) were retrieved from the China Meteorological Data Service Center and collected over the same time period of time as the ILI data (Fig. 1). The meteorological variables were average, maximum, minimum air temperature (°C), vapor pressure (0.1 hPa) and relative humidity (%).

### Statistical analysis

Surveillance data of ILI cases and meteorological variables were exported to Microsoft Excel 2003, continuous variables are expressed as the mean ± standard deviation or median (*P*_25_, *P*_75_), while categorical variables are described by absolute and relative frequencies.

The Spearman correlation analyses was performed to explore the correlations between the meteorological factors. Generalized additive model was used to seek the relationship between the number of ILI cases and the meteorological factors. GAM proposed by Hastie & Tibshirani (1990) is a generalized linear model in which part of the linear predictor is specified in terms of sum of smooth functions of predictor variables.

The basic form of GAM applied in the present study is expressed using the following Eq. (1).

(1) \begin{eqnarray*}g[E({Y}_{i})]={\beta }_{0}+\sum _{i=1}^{m}{s}_{i}({x}_{i-l})+{\varepsilon }_{i}.\end{eqnarray*}

In the present study, *Y*_*i*_ is the count of weekly number of ILI, *β*_0_ denotes the intercept, *i* indicates the calendar week, multiple smoothing parameter estimation was made based on the basis of Poisson distribution *g*, *s*_*i*_(*x*_*i*−*l*_) denotes a smooth function of meteorological variables *x*_*i*−*l*_, ε_*i*_ is error. Lag time *l* was determined by the minimum AICs. The lag effect of weather for ILI cases was explored for one and two weeks according to previous literature (Davis, Rossier & Enfield, 2012; Zhao et al., 2018).

The Spearman correlation analyses were conducted by using the software IBM SPSS Statistics 21.0 for windows (IBM, Asian Analytics Shanghai) and the mgcv package in R software was used to construct the GAM models (Code S1).

## Results

### Description of ILI surveillance dataset

**Figure 2:**
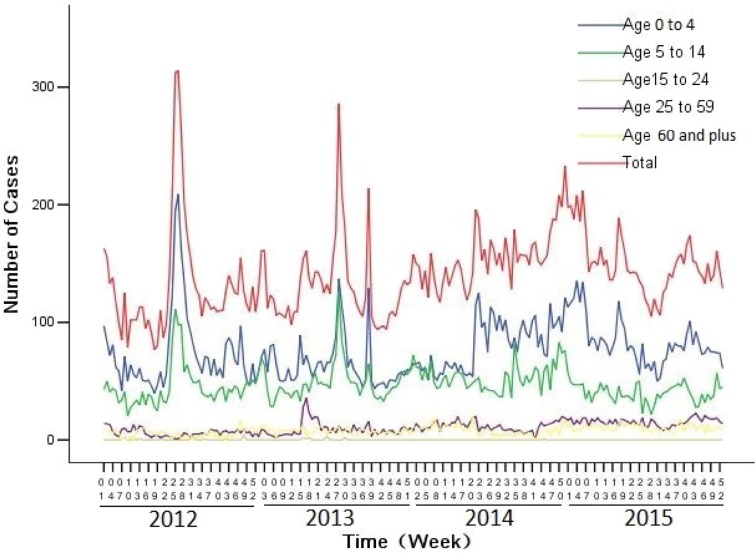
Number of influenza-like illness cases by age from 2012 to 2015 in Huludao, China. Each data line indicates the temporal distribution of ILI cases in different age groups.

A total of 29 622 weekly reported ILI cases from 2012 to 2015 in Huludao city were collected (Fig. 2, Table 1, Dataset S1). During the study period, the total percentage of children with ILI was approximately 86.77%.

### Spearman correlation coefficients between meteorological variables

The correlation analysis between the meteorological factors indicates that average air temperature, maximum air temperature, minimum air temperatures and vapor pressure are strongly correlated with each other, and correlated with relative humidity moderately (Table 2).

**Table 1:** Descriptive of number of ILI cases in different age groups from 2012 to 2015 in Huludao, China.

**Age group (years)**	**Minimum**	**Maximum**	**Mean**	**Std. deviation**	**Sum**	**Percentage (%)**
0–4	40	209	75.89	25.59	15,861	53.5
5–14	21	126	47.10	14.49	9,843	33.2
15–24	0	4	0.12	0.51	26	0.1
25–59	0	36	10.69	5.67	2,235	7.6
60 and elder	0	20	7.93	3.82	1,657	5.6
Total	77	314	141.73	36.25	29,622	100.00

**Table 2:** Spearman correlation coefficients between meteorological variables from 2012 to 2015 in Huludao, China.

	Average air temperature	Maximum air temperature	Minimum air temperature	Vapor pressure	Relative humidity
Average air temperature	1.00				
Maximum air temperature	0.99^*^	1.00			
Minimum air temperature	0.99^*^	0.97^*^	1.00		
Vapor Pressure	0.97^*^	0.94^*^	0.98^*^	1.00	
Relative Humidity	0.54^*^	0.48^*^	0.59^*^	0.71^*^	1.00

**Notes.**

* indicates that the correlation is statistically significant at the 0.01 level (two-tailed).

### Lag effect selection for individual meteorological variable

The lag time for average air temperature, maximum air temperature, minimum air temperature, vapor pressure and relative humidity are 2, 2, 1, 1 and 0 weeks, respectively (Table 3).

**Table 3:** Lag effect selection for individual meteorological variable according to Akaike information criterion (lower AIC indicates better model fit).

Variables	Lag 0 week	Lag 1 week	Lag 2 weeks
Average air temperature	2940.98	2892.84	2890.21
Maximum air temperature	3095.34	3055.03	3012.91
Minimum air temperature	2916.58	2871.81	2879.52
Vapor pressure	2940.17	2909.07	2933.02
Relative humidity	3061.01	3068.42	3109.19

**Notes.**

AIC Akaike information criterion

### Summary for individual meteorological variable and two explanatory variables for GAM models

In the single variable models, the adjusted *R*^2^ for average air temperature, maximum air temperature, minimum air temperature, vapor pressure and relative humidity is 11.5%, 4.7%, 13.2%, 12.4%, 3.2%, respectively, and the models can each explain 16.5%, 9.53%, 18.0%, 15.9%, 7.7% of the deviance of the number of ILI cases (Table 4). The effective degree of freedom for each variable is greater than 8, indicating the complex spline relationship between meteorological variables and the occurrence of ILI cases. Minimum air temperature and relative humidity were included in the two variable model, because minimum air temperature is the best explanatory temperature index among the three temperature indexes, relative humidity is the least relevant explanatory variable with the other four meteorological variable. The two variable model could explain 23.2% of the deviance.

**Table 4:** Summary for individual meteorological variable and two explanatory variables for GAM models.

Individual explanatory variable	Effective degree of freedom	Reference number of degree of freedom	Chi-square	*P*-value	Adjusted *R*^2^ (%)	Explained deviance (%)
Average air temperature (Lag 2 weeks)	8.90	9.00	292.2	<2 × 10^−16^	11.5	16.5
Maximum air temperature (Lag 2 weeks)	8.61	8.96	163.7	<2 × 10^−16^	4.7	9.53
Minimum air temperature (Lag 1 week)	8.88	9.00	319.5	<2 × 10^−16^	13.2	18.0
Vapor pressure (Lag 1 week)	8.57	8.94	290.1	<2 × 10^−16^	12.4	15.9
Relative humidity (Lag 0 week)	8.65	8.97	137.9	<2 × 10^−16^	3.2	7.7
Two Explanatory variables						
Minimum air temperature (Lag 1 week)	8.85	8.99	267.0	<2 × 10^−16^	14.8	23.2
Relative humidity (Lag 0 week)	8.67	8.97	91.2	9.26 × 10^−16^		

**Notes.**

GAM Generalized additive model

### ILI activity and Relative risk of the meteorological variables

**Figure 3:**
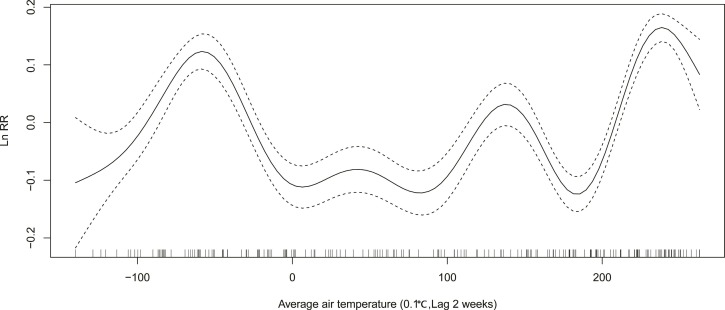
Relationship between ILI activity and average air temperature obtained from smooth functions in Generalized additive model, 2012–2015 in Huludao, China. The solid line indicates the relative risk’s natural logarithm of average air temperature, and the two dotted lines represent the lower and upper limit of 95% confidence interval.

**Figure 4:**
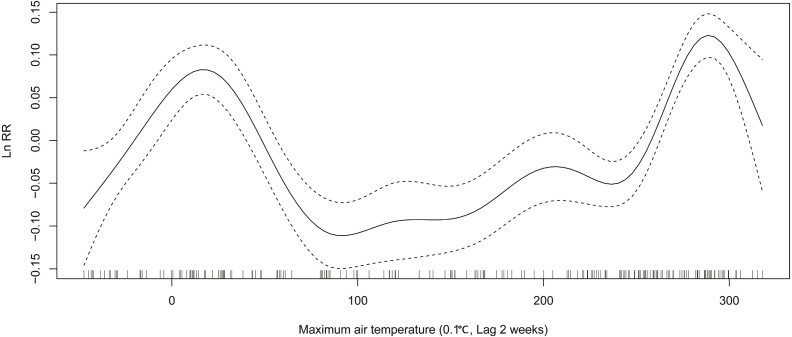
Relationship between ILI activity and maximum air temperature obtained from smooth functions in Generalized additive model, 2012–2015 in Huludao, China. The solid line indicates the relative risk’s natural logarithm of maximum air temperature, and the two dotted lines represent the lower and upper limit of 95% confidence interval.

**Figure 5:**
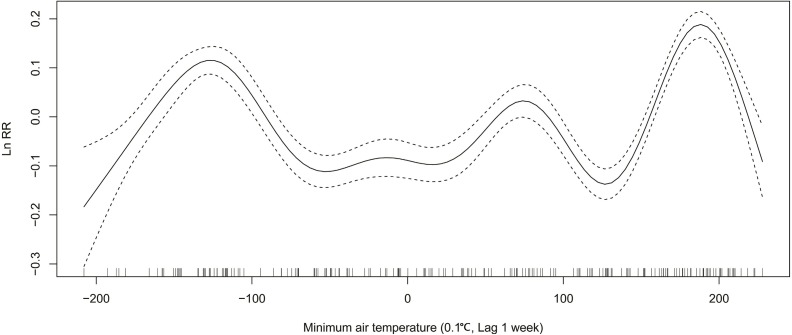
Relationship between ILI activity and minimum air temperature obtained from smooth functions in Generalized additive model, 2012–2015 in Huludao, China. The solid line indicates the relative risk’s natural logarithm of minimum air temperature, and the two dotted lines represent the lower and upper limit of 95% confidence interval.

**Figure 6:**
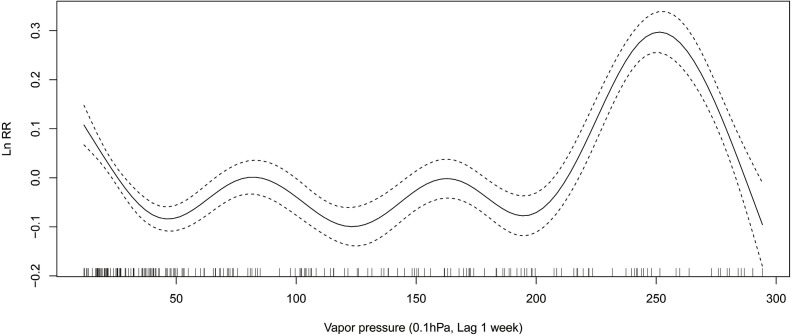
Relationship between ILI activity and vapor pressure obtained from smooth functions in Generalized additive model, 2012–2015 in Huludao, China. The solid line indicates the relative risk’s natural logarithm of vapor pressure, and the two dotted lines represent the lower and upper limit of 95% confidence interval.

**Figure 7:**
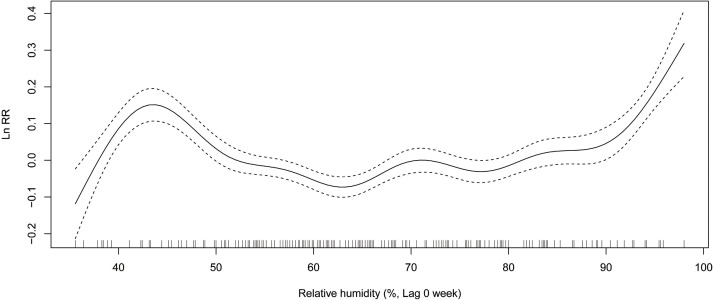
Relationship between ILI activity and relative humidity obtained from smooth functions in Generalized additive model, 2012–2015 in Huludao, China. The solid line indicates the relative risk’s natural logarithm of relative humidity, and the two dotted lines represent the lower and upper limit of 95% confidence interval.

In winter, relative risk (RR) of ILI decreases from 1.14 (exp(0.13)) to 0.90 (exp(−0.11)), when the average air temperature changes from −4 °C to 1 °C (Fig. 3). When the maximum air temperature changes from 2 degrees to 8 degrees, RR decreases from 1.08 (exp(0.08)) to 0.88 (exp(−0.13)) (Fig. 4). When the minimum air temperature changes from −13 °C to −6 °C, RR decreases from 1.11 (exp(0.10)) to 0.90 (exp(−0.10)) (Fig. 5). RR decreases from 1.11 (exp(0.10)) to 0.92 (exp(−0.08)) as the vapor pressure changes from 10 (0.1 hPa) to 45 (0.1 hPa) (Fig. 6). When relative humidity changes from 43% to 64%, RR decreases from 1.15 (exp(0.14)) to 0.95 (exp(−0.05)) (Fig. 7).

In summer, RR increases from 0.88 (exp(−0.13)) to 1.16 (exp(0.15)), when the average air temperature changes from 18 °C to 24 °C (Fig. 3). When the maximum air temperature changes from 24 °C to 29 °C, RR increases from 0.95 (exp(−0.05)) to 1.14 (exp(0.13)) (Fig. 4). RR increases from 0.88 (exp(−0.13)) to 1.21 (exp(0.19)), when the minimum temperature changes from 13 °C to 18 °C (Fig. 5). When the vapor pressure changes from 195 (0.1 hPa) to 255 (0.1 hPa), the RR value increases from 0.94 (exp(−0.06)) to 1.32 (exp(0.28)) (Fig. 6). RR increases from 1.00 (exp(0.00)) to 1.35(exp(0.30)), when relative humidity changes from 76% to 98% (Fig. 7).

## Discussion

The associations between the climatic data and the incidence of ILI cases have been observed in many temperate regions, while the strength and the direction of these associations were location-dependent (Shaman & Karspeck, 2012; Thai et al., 2015). We identified the distinct seasonality for the incidence of ILI cases in the study area, two peaks in a year, one in summer and the other in winter. Consistent with other findings (Gordon et al., 2009; Guo et al., 2012; Oliveira et al., 2016; Wijngaard et al., 2012), the children aged under 14 years old were highly vulnerable to ILI infections and constituted more than 85% of the ILI cases, which provided baseline information for the allocation of health resources and also indicated that health education programs need to be arranged particularly for children (De Lange et al., 2013; Weng et al., 2015).

Generally, the incidence of ILI cases is considered as time series, the correlation may exist between the number of cases, thus these data is often analyzed by adopting the generalized additive model, generalized estimated equation and other similar methods (Azman et al., 2013; Liang et al., 2018; Nielsen et al., 2011; Shaman et al., 2013; Vega et al., 2015). The GAMs constructed in the present study indicated that low air temperature and low relative humidity (cold and dry weather condition) played a considerable role in the epidemic pattern of ILI cases. The role of a cold and dry weather on influenza spread has also been highlighted from laboratory and epidemiological studies in different countries (Emukule et al., 2016; Jusot, Adamou & Collard, 2012; Thai et al., 2015).

The temporal variables were also considered and analyzed in the present study, we demonstrated that the significant link between the weather variation in a short time and ILI activity in warm temperate climates. The relationship between the climatic data and the incidence of ILI cases have been explored by other researchers using the correlation analysis, they indicated that, the weather pattern showed a statistically clear influence on ILI cases and it strongly correlated with humidity at a lag of one week to one month, while temperature had a weaker correlation (Nimbalkar & Tripathi, 2016; Roussel et al., 2016). However, this kind of phenomenon was not found in the present study located in temperate regions. The difference of the length of lag could be partially explained by the data on a monthly basis in Thailand (Nimbalkar & Tripathi, 2016), while the data collected in the present study was on a weekly basis. Large-scale analyses revealed that no single climatic variable could capture the complexity of influenza seasonality patterns (Tamerius et al., 2013), while minimum temperature, humidity, and precipitations could help distinguish influenza seasonality patterns (Yu et al., 2013).

The present study, however, had several limitations. The first limitation was that the high proportion (86.77%) of children among all the collected ILI cases. Although all the ILI cases have been considered and analyzed in the present study, the result could mainly indicate the relationship between the climatic data and ILI cases among children aged under 14 years. Thus, more attention should be paid to the extrapolation of the present study’s results. The second limitation is that this was a one-site study, only the ILI cases from Huludao Municipal Center Hospital were collected. The limitation of surveillance coverage range may have an impact on the estimate of the epidemic pattern of ILI cases for the whole city. Further training of more experienced clinicians in this field is required to strengthen the sensitivity of the surveillance system. The third limitation is that all the climatic variables considered in the present study were taken outdoors, so the relationship mentioned here may not imply the same effect indoors (Tamerius et al., 2017). In addition, there are some other climatic factors involved in the transmission of influenza that may not have been discussed in the present study.

## Conclusions

In summary, the main findings of this study were that low air temperature and low relative humidity (cold and dry weather condition) played a considerable role in the epidemic pattern of ILI cases. The present study first analyzed the relationship between meteorological factors and ILI cases with special consideration of the length of lag period in Huludao, China. It highlighted the possibility that the trend of ILI activity could be possibly predicted by the variation of climatic variables. The mechanism of action of fluctuation in the climatic variables needs to be further investigated.

##  Supplemental Information

10.7717/peerj.6919/supp-1 Supplemental Information 1 Software R code in the analysis Raw data and take the average air temperature as the example.

10.7717/peerj.6919/supp-2 Supplemental Information 2 Meteorological factors and Huludao ILI cases from 2012–2015 Raw data analyzed in the study.

## Additional Information and Declarations

### Data Availability

The following information was supplied regarding data availability: The software R codes adopted in the article and the raw data for meteorological factors and Huludao ILI cases from 2012 to 2015 are available in the Supplemental Files.
